# *In Vitro* Reactivation of Replication-Competent and Infectious HIV-1 by Histone Deacetylase Inhibitors

**DOI:** 10.1128/JVI.02359-15

**Published:** 2016-01-28

**Authors:** Riddhima Banga, Francesco Andrea Procopio, Matthias Cavassini, Matthieu Perreau

**Affiliations:** a Service of Immunology and Allergy, Lausanne University Hospital, University of Lausanne, Lausanne, Switzerland; b Service of Infectious Diseases, Lausanne University Hospital, University of Lausanne, Lausanne, Switzerland

## Abstract

The existence of long-lived HIV-1-infected resting memory CD4 T cells is thought to be the primary obstacle to HIV-1 eradication. In the search for novel therapeutic approaches that may reverse HIV-1 latency, inhibitors of histone deacetylases (HDACis) have been tested to reactivate HIV-1 replication with the objective of rendering HIV-1-infected cells susceptible to elimination either by HIV-specific CD8 T cells or through virus-mediated cytopathicity. In the present study, we evaluated the efficiency of HDACis to reactivate HIV-1 replication from resting memory CD4 T cells isolated from aviremic long-term-treated HIV-1-infected subjects. We demonstrate that following prolonged/repeated treatment of resting memory CD4 T cells with HDACis, HIV-1 replication may be induced from primary resting memory CD4 T cells isolated from aviremic long-term-treated HIV-1-infected subjects. More importantly, we demonstrate that HIV-1 reactivated in the cell cultures was not only replication competent but also infectious. Interestingly, givinostat, an HDACi that has not been investigated in clinical trials, was more efficient than vorinostat, panobinostat, and romidepsin in reversing HIV-1 latency *in vitro*. Taken together, these results support further evaluation of givinostat as a latency-reversing agent (LRA) in aviremic long-term-treated HIV-1-infected subjects.

**IMPORTANCE** The major barrier to HIV cure is the existence of long-lived latently HIV-1-infected resting memory CD4 T cells. Latently HIV-1-infected CD4 T cells are transcriptionally silent and are therefore not targeted by conventional antiretroviral therapy (ART) or the immune system. In this context, one strategy to target latently infected cells is based on pharmacological molecules that may force the virus to replicate and would therefore render HIV-1-infected cells susceptible to elimination either by HIV-specific CD8 T cells or through virus-mediated cytopathicity. In this context, we developed an experimental strategy that would allow the evaluation of latency-reversing agent (LRA) efficiency *in vitro* using primary CD4 T cells. In the present study, we demonstrate that HDACis are potent inducers of replication-competent and infectious HIV-1 in resting memory CD4 T cells of long-term ART-treated patients and identify givinostat as the most efficient LRA tested.

## INTRODUCTION

The existence of long-lived HIV-1-infected resting memory CD4 T cells represents the primary obstacle to HIV-1 eradication (1–6). In this regard, it has been hypothesized that latency-reversing agents (LRAs) that may reactivate HIV-1 replication from latently infected cells may render HIV-1-infected cells susceptible to elimination either by HIV-specific CD8 T cells or through virus-mediated cytopathicity (7). The use of *in vitro* models of HIV-1 latency has contributed to evaluate LRA efficiency in the reactivation of HIV-1 replication (8–14). However, such types of assays also face some limits inherent in the clonality of the HIV-1 integration site (8–11) or the frequency of latently infected cells (11–14). To circumvent this caveat, several groups have evaluated the efficiency of LRAs on primary resting CD4 T cells using various strategies, including the “classical,” “modified,” or “enhanced” viral outgrowth assay (VOA) (15–17).

Using a modified version of the “classical” VOA, David Margolis' group first showed that valproic acid induced outgrowth of HIV from resting CD4 T cells of aviremic patients at concentrations achievable *in vivo*, thus demonstrating that histone deacetylase inhibitors (HDACis) could reactivate HIV-1 replication from resting CD4 T cells *in vitro* (17). More recently, John Mellors' and Robert Siliciano's groups have evaluated the efficiency of LRAs on primary resting memory CD4 T cells and have underscored the difficulty of reactivating HIV-1 replication in primary resting memory CD4 T cells (15, 16). On the basis of these results, it was concluded that HDACis may have limited effectiveness in the reactivation of replication-competent HIV-1 in primary resting memory CD4 T cells (15), unless a combination of mechanistically distinct LRAs is used (18).

The relative lack of efficacy of LRAs to reactivate HIV-1 replication *in vitro* contrasts with the findings of clinical studies showing promising results on the ability of HDACis (via single-dose or multidose administration) such as vorinostat, romidepsin, and panobinostat to increase cell-associated RNA and more importantly to induce transient blips in viremia in otherwise aviremic antiretroviral therapy (ART)-treated subjects (19, 20, 31). In addition, a recent study from Dar et al. postulated that increasing baseline transcription noise can enhance the probability of successful viral release from HIV-infected cells (21). In this context, we hypothesized that repeated/prolonged treatment of resting memory CD4 T cells with HDACis in the presence of increased baseline transcription noise may reactivate HIV-1 replication from primary resting memory CD4 T cells isolated from aviremic long-term-treated HIV-1-infected subjects.

Therefore, in the present study, we used a modified VOA that integrates a number of strategies that may potentiate the therapeutic effects of HDACis and create better experimental conditions for amplification of HIV-1 replication *in vitro*. These include (i) increased duration of treatment of resting memory CD4 T cells with HDACis to mimic the regimens associated with the better therapeutic effects of these drugs when administered to patients and (ii) use of allogeneic (irradiated or not) CD8-depleted blood mononuclear cells from HIV-1-uninfected subjects that may provide large numbers of novel CD4 T-cell targets to amplify HIV-1 replication *in vitro* and/or increase baseline transcription noise, which in turn may enhance the probability of successful viral release from cells exposed to HDACis (21).

We demonstrate that following prolonged/repeated treatment of resting memory CD4 T cells with HDACis, HIV-1 replication can be induced from primary resting memory CD4 T cells isolated from aviremic long-term-treated HIV-1-infected subjects. The use of allogeneic nonirradiated blood mononuclear cells appears to have a secondary effect since it was associated only with a minor effect on HIV-1 replication. More importantly, we demonstrate that HIV-1 reactivated in the cell cultures was not only replication competent but also infectious. Interestingly, givinostat, an HDACi that has not been investigated in clinical trials, was more efficient than vorinostat, panobinostat, and romidepsin in reversing HIV-1 latency *in vitro*.

## MATERIALS AND METHODS

### Ethics statement.

The present study was approved by the Institutional Review Board of Lausanne University Hospital, University of Lausanne (i.e., Centre Hospitalier Universitaire Vaudois), and all subjects gave written informed consent.

### Study group, ethics statement, and cell isolation.

Ten HIV-1-infected adult volunteers (Table 1) and six HIV-uninfected subjects were enrolled in the present study. No statistical method was used to predetermine the sample size. The sample size was estimated based on a previously published study (15). As inclusion criteria, only patients under antiretroviral therapy for more than 2 years with undetectable HIV-1 viremia (<20 HIV-1 RNA copies/ml) were enrolled in the study. As exclusion criteria, patients experiencing blips of viremia (>50 HIV-1 RNA copies per ml of plasma) within the last 12 months were not enrolled. Leukapheresis and blood samples were obtained at the local blood bank (Centre de Transfusion Sanguine [CTS], Lausanne, Switzerland). Blood mononuclear cells were isolated as previously described (22). Since the inclusion criteria were the same for the 10 patients studied, there was no need for randomization or blinding.

**TABLE 1:** Characteristics of the study group

Subject no.	Age (yr)	Sex	Duration of HIV infection (yr)	Result at enrollment		Time on HAART (yr)^*a*^	HAART regimen^*b*^	No. of:		
				CD4 cell count (cells/μl)	Viral load (copies/ml)			Integrated HIV-1 DNA copies/million resting memory CD4 T cells	HIV RNA copies/million resting CD4 memory T cells^*c*^	Infectious units/million resting memory CD4 T cells^*d*^
1	47	M	5	760	<20	5	ETR, FTC, TDF	33	4.17	0.35
5	41	F	9	1,370	<20	6	TDF, FTC, ATV/r	5,951	29	0.77
6	51	M	6	391	<20	6	FTC, TDF, ETV	7	0.35	<0.35
7	36	M	6	760	<20	3	ABC, 3TC, ATV/r	1,765	2.05	<0.35
8	41	M	15	323	<20	6	ABC, 3TC, EFV	1,170	5.13	<0.35
10	49	M	8	543	<20	8	TDF, FTC, EFV	958	39.39	1.19
12	29	F	8	473	<20	2	ABC, 3TC, DRV/r	1,179	4.28	0.35
14	37	M	6	1,267	<20	2	TDF, FTC, EFV	972	29	0.77
16	49	M	25	549	<20	2	ABC, 3TC, EFV	14,309	16.47	1.31
18	45	M	3	376	<20	2	TDF, FTC, EFV	2,074	1.81	<0.35

a HAART, highly active antiretroviral therapy.

b ETR, etravirine; FTC, emtricitabine; TDF, tenofovir disoproxil fumarate; ATV/r, atazanavir boosted with ritonavir; 3TC, lamivudine; ABC, abacavir; DRV/r, darunavir boosted with ritonavir; EFV, efavirenz.

c Designated “RUPM” in the text and selected figures.

d Designated “IUPM” in the text and selected figures.

### Reagents and cell culture.

Vorinostat (Merck Research Laboratory, USA), romidepsin (Cellgene, USA), panobinostat (Novartis, Switzerland), givinostat (Italfarmaco; Italy), belinostat (Topotarget and Spectrum Pharmaceuticals, Denmark), and bryostatin were obtained from Hölzel Diagnostika (Germany) and resuspended in dimethyl sulfoxide (DMSO). Cells were cultured in RPMI (Gibco, Life Technologies) containing 10% heat-inactivated fetal bovine serum (FBS) (Institut de Biotechnologies Jacques Boy), 100 IU/ml penicillin, and 100 μg/ml streptomycin (Bio Concept).

### Antibodies.

The following antibodies were used. Allophycocyanin (APC)-H7-conjugated anti-CD3 (clone SK7), peridinin chlorophyll protein (PerCP)-Cy5.5-conjugated anti-CD8 (clone SK1), Pacific Blue (PB)-, fluorescein isothiocyanate (FITC)-, or phycoerythrin (PE)-CF594-conjugated anti-CD4 (clone RPA-T4), V450-conjugated anti-HLA-DR (clone G46-6), PE-Cy7-conjugated anti-CD25 (clone M-A251), PerCP-Cy5.5-conjugated anti-CD69 (clone L78), purified coating anti-CD3 (clone UCHT1), and anti-CD28 (clone CD28.2) monoclonal antibodies (MAbs) were purchased from BD (Becton Dickinson, USA) or from Biolegend (Switzerland). PE-conjugated anti-acetyl H3 and H4 were from Merck Millipore (USA), and electron-coupled dye (ECD)-conjugated anti-CD45RA (clone 2H4) was from Beckman Coulter (USA).

### Flow cytometry.

Data were acquired on a 4-laser LSR Sorp flow cytometer (405, 488, 532, and 633 nm) and were analyzed using FlowJo v9.4.11 (Treestar, Inc., Ashland, OR). At least 100,000 events were acquired for each sample.

### Sorting of resting memory CD4 T cells.

Cryopreserved blood mononuclear cells were thawed, and CD4 T cells were enriched using EasySep human CD4 T-cell enrichment kit (StemCell Technologies, USA). CD4 T cells were then stained with an Aqua LIVE/DEAD stain kit (4°C, 15 min) and then with anti-CD4–FITC, anti-CD45RA–ECD, anti-HLA-DR–PB, anti-CD25–PE-Cy7, and anti-CD69–PerCp-Cy5.5 (4°C, 25 min) and viable resting memory (CD4^+^ CD45RA^−^ CD25^−^ CD69^−^ HLA-DR^−^) CD4 T-cell populations were sorted using a FACSAria fluorescence-activated cell sorter (Becton Dickinson). In all sorting experiments, the grade of purity of the sorted cell populations was >97%.

### Integrated HIV-1 DNA quantification.

Resting memory CD4 T cells were sorted as described above and lysed using lysis buffer (10 mM Tris-HCl, pH 8.0, 50 nM KCl, 400 μg/ml proteinase K [Invitrogen]), and integrated HIV-1 DNA and CD3 gene copy numbers were determined using a cross-clade ultrasensitive nested Alu PCR, as previously described (23). The frequency of HIV-1 integrated DNA per million cells was calculated as previously described (23).

### Modified viral outgrowth assay.

Different cell concentrations (5-fold limiting dilutions of 5 × 10^5^, 10^5^, 2 × 10^4^, and 4 × 10^3^ cells) of sorted viable resting memory CD4 T cells (CD4^+^ CD45RA^−^ CD25^−^ CD69^−^ HLADR^−^) from HIV-1-infected subjects (Table 1) were cultured (5 replicates per condition) in complete RPMI with allogeneic fresh CD8-depleted blood mononuclear cells (10^6^ cells/ml) from HIV-uninfected subjects in the presence or absence (negative control) of an HDACi: i.e., vorinostat (400 nM), romidepsin (5 nM), panobinostat (15 nM), givinostat (400 nM), or belinostat (400 nM). As a positive control, cells were stimulated for 3 days with anti-CD3/CD28 MAb-coated plates (10 μg/ml). In some experiments, sorted viable resting memory CD4 T cells from HIV-1-infected subjects were exposed to (i) givinostat (400 nM) and/or bryostatin (10 nM) for 18 h or (ii) givinostat (400 nM) for 14 days and/or anti-CD3/CD28 MAbs. In some experiments, allogeneic CD8-depleted peripheral blood mononuclear cells (PBMCs) from HIV-1-negative subjects were irradiated (40 Gy) or replaced by autologous irradiated (40 Gy) CD8-depleted PBMCs or allogeneic irradiated (40 Gy) CD8-depleted PBMCs. All cell conditions involved culture in complete RPMI supplemented with interleukin-2 (IL-2) (50 U/ml) and IL-7 (10 ng/ml) for 14 days. Medium was replaced at day 5 and resupplemented with HDACi and cytokines. Supernatants were collected at day 14. The presence of P24 antigen was assessed by ECL Cobas HIV Ag (Roche; Switzerland). One enhanced chemiluminescence (ECL) unit corresponds to about 22 pg/ml. The presence of HIV-1 RNA was assessed by the Cobas AmpliPrep/TaqMan HIV-1 test (Roche, Switzerland) or by the Abbott RealTime HIV-1 assay (Abbott, USA) following 1/10 medium dilution in basement matrix buffer (Ruwag Handels AG). The numbers of replication-competent units per million cells (RUPM) (24) and infectious units per million cells (IUPM) (15) were calculated by conventional limiting dilution methods using extreme limiting dilution analysis (http://bioinf.wehi.edu.au/software/elda/) (25).

### *In vitro* HIV-1 infection assay.

CD8-depleted blood mononuclear cells isolated from HIV-uninfected donors were activated for 48 h with anti-CD3/CD28 microbeads (Miltenyi) in complete RPMI medium supplemented with IL-2 (50 U/ml). Activated allogeneic CD8-depleted blood mononuclear cells (10^6^ cells/ml) from HIV-1-uninfected donors were washed and exposed (6 h, 37°C, 5% CO_2_) to 100 μl P24^+^ supernatants collected at day 14 following various modified VOA conditions (anti-CD3/CD28 MAbs or HDACi treatments). Following 6 h of exposure, cells were washed twice with complete medium and cultured for an additional 10 days in complete RPMI medium. The presence of infectious HIV-1 particles was determined in the culture supernatants at days 0, 3, and 10 postinoculation as assessed by P24 production assay using ECL.

### Assessment of CD4 T-cell viability.

CD4 T cells isolated from 3 HIV-uninfected individuals were exposed or not to vorinostat (400 nM), romidepsin (5 nM), panobinostat (15 nM), givinostat (400 nM), or belinostat (400 nM) in the presence of allogeneic CD8-depleted PBMCs isolated from HIV-uninfected subjects. After 24, 48, 96, and 144 h, cells were stained with the Aqua LIVE/DEAD stain kit (4°C, 15 min), washed, and stained with anti-CD3 APC-H7, anti-CD4-FITC, and annexin V-APC (4°C, 20 min). Cells were washed with annexin buffer, and the percentages of CD3^+^ CD4^+^ Aqua^+^ and CD3^+^ CD4^+^ annexin^+^ cells were assessed by flow cytometry.

### Proliferation inhibition assay.

Blood mononuclear cells were stained with 0.25 μM 5,6-carboxyfluorescein succinimidyl ester (CFSE [Molecular Probes, USA]) as previously described (22) and stimulated for 6 days with anti-CD3/CD28 MAb-coated plates (10 μg/ml), or left unstimulated, in the presence or in the absence of HDACi: vorinostat (400 nM), romidepsin (5 nM), panobinostat (15 nM), givinostat (400 nM), or belinostat (400 nM). Cells were then washed and stained (30 min, 4°C) with anti-CD3–APC-H7 and anti-CD4–PE-CF594, and the percentage of viable cells was assessed using the Vivid LIVE/DEAD stain kit (Invitrogen). Proliferating CD4 T cells were defined as viable CD3^+^ CD4^+^ CFSE^low^ cells.

### Evaluation of HDACi-induced histone H3 and H4 acetylation.

Freshly isolated blood mononuclear cells were exposed or not to HDACi (i.e., vorinostat [400 nM], romidepsin [5 nM], panobinostat [15 nM]), givinostat [400 nM], or belinostat [400 nM]) for 24 h, and histone H3 and H4 acetylation was assessed as previously described (26). Briefly, cells were stained with the Aqua LIVE/DEAD stain kit (4°C, 20 min), permeabilized (4°C, 1 h) with a Foxp3 fixation/permeabilization kit (eBioscience), blocked (4°C, 10 min) with FBS, and washed and stained (4°C, 25 min) with anti-CD3–APC-H7, anti-CD8–PerCp-Cy5.5, anti-CD4–PB, and anti-acetyl H3–PE or anti-acetyl H4–PE. Expression of acetyl histone H3 and H4 in CD4 T-cell population was assessed by flow cytometry.

### Assessment of level of expression of activation marker and proliferation capacity of purified resting memory CD4 T cells exposed to allogeneic CD8-depleted mononuclear cells.

Sorted viable resting memory CD4 T cells (CD4^+^ CD45RA^−^ CD25^−^ CD69^−^ HLADR^−^) from HIV-1-uninfected subjects (*n* = 3) were labeled with CFSE and cultured with allogeneic fresh CD8-depleted blood mononuclear cells (10^6^ cells/ml) from HIV-1-uninfected subjects or remained unexposed (negative control). As a positive control, cells were stimulated for 3 days with anti-CD3/CD28 MAb-coated plates (10 μg/ml). All cell conditions were cultured in complete RPMI medium supplemented with IL-2 (50 U/ml) and IL-7 (10 ng/ml) for 14 days. At days 0, 1, 2, 3, and 6, cells were washed and stained (30 min, 4°C) with anti-CD3–APC-H7, anti-CD4–PE-CF594, anti-HLA-DR–PB, anti-CD25–PE-Cy7, and anti-CD69–PerCp-Cy5.5, and the percentage of viable cells was assessed using the Vivid LIVE/DEAD stain kit (Invitrogen). Proliferating CD4 T cells were defined as viable CD3^+^ CD4^+^ CFSE^low^ cells.

### Statistical analyses.

Statistical significance (*P* values) was obtained either using a two-tailed chi-square analysis for comparison of positive proportions or by one-way analysis of variance (ANOVA) (Kruskal-Wallis test) followed by a Wilcoxon matched-pair two-tailed signed rank test for multiple comparisons. When required, Bonferroni's correction was applied for multiple comparisons. Finally, Spearman's rank test was used for correlations.

## RESULTS

### Experimental strategy to evaluate the efficiency of HDACis to reverse HIV-1 latency in resting memory CD4 T cells isolated from long-term-treated HIV-1-infected individuals.

**FIG 1:**
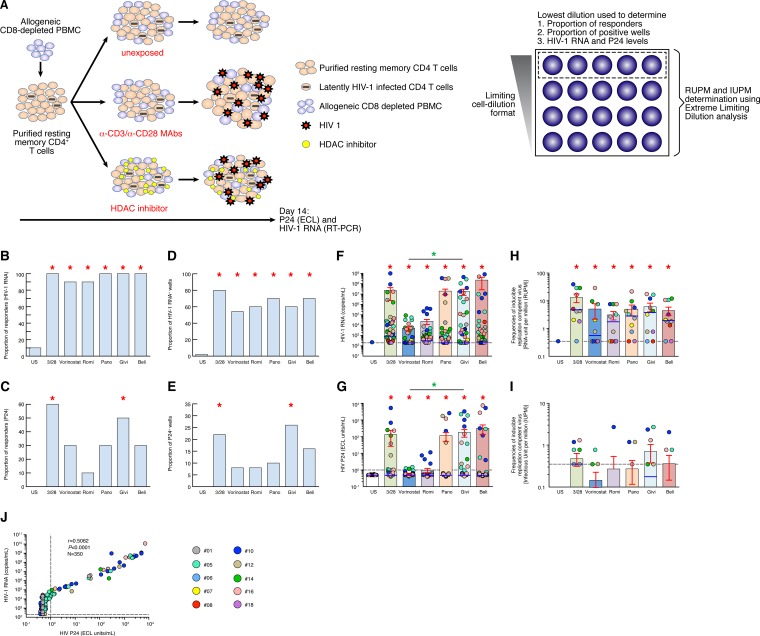
HDACis efficiently reactivate HIV-1 replication from latently infected resting memory CD4 T cells isolated from long-term-treated HIV-1-infected subjects. (A) Schematic representation of the modified VOA. (B) Proportion of responders to HDACis treatment based on the detection of HIV-1 RNA (*n* = 10; 5 replicates per condition). Individuals having at least one replicate with detectable HIV-1 RNA (≥200 HIV-1 RNA copies/ml) are indicated as “responders” for the condition tested. (C) Proportion of responders to HDACi treatment based on the detection of P24 (*n* = 10; 5 replicates per condition). Individuals having at least one replicate with detectable P24 (≥1 ECL unit/ml) are indicated as “responders” for the condition tested. (D) Proportion of HIV-1 RNA-positive wells induced following HDACis treatment (*n* = 10; 5 replicates per condition). Wells with detectable HIV-1 RNA (≥200 HIV-1 RNA copies/ml) are indicated to as HIV-1 RNA positive for the condition tested. (E) Proportion of P24-positive wells induced following HDACis treatment (*n* = 10; 5 replicates per condition). Wells with detectable P24 (≥1 ECL unit/ml) are indicated as P24 positive for the condition tested. (F) Levels of HIV-1 RNA copies per milliliter induced following HDACis treatment (*n* = 10; 5 replicates per condition). (G) Levels of P24 (ECL units per milliliter) induced following HDACis treatment (*n* = 10; 5 replicates per condition). (H and I) Frequencies of inducible replication-competent virus as measured by replication-competent (RNA) units per million (RUPM) (H) or as measured by infectious units per million (IUPM) (I). (J) Correlation between P24 and HIV-1 RNA levels (*n* = 350; 10 subjects, 7 conditions, 5 replicates per condition). Panels B to G and J were generated using the 5 replicates of the lowest dilution of cells (5 × 10^5^ cells) of all conditions by modified VOA. Subjects were color coded, and each color corresponds to a subject (F to J). Histograms correspond to the mean (B to I), and red error bars correspond to the standard error of the mean (SEM) (F to I). Blue lines correspond to the median (F to I). Red asterisks indicate statistical significance compared to unstimulated or unexposed (US) (*P* < 0.05). Green asterisks indicate statistical significance compared to vorinostat (*P* < 0.05). romi, romidepsin; pano, panobinostat; givi, givinostat; beli, belinostat. Statistical significance (*P* values) was obtained using two-tailed chi-square analysis for comparison of positive proportions (B to E), by one-way ANOVA (Kruskal-Wallis test) followed by Wilcoxon matched-pair two-tailed signed rank test (F to I), or by using Spearman's rank correlations (J). Bonferroni's correction was applied for multiple comparisons.

The modified VOA is schematically outlined in Fig. 1A. As a positive control, resting memory CD4 T cells were stimulated with anti-CD3/CD28 MAbs for 3 days in the presence of IL-2 and IL-7. The presence of HIV-1 RNA and P24 in the culture supernatants were measured using validated diagnostic assays (Fig. 1A). It is important to underscore that the concentrations of HDACis used in the modified VOA assay corresponded to the clinical doses used in patients. The proportion of responders, proportion of positive wells, and HIV-1 RNA and P24 levels were generated using the 5 replicates of the lowest dilution of cells (5 × 10^5^ cells/condition) in the modified VOA (Fig. 1A), while the limiting dilution format was used to evaluate the frequencies of inducible replication-competent virus from latently HIV-1-infected cells. The frequencies of cells containing replication-competent virus were assessed by (i) the detection of HIV RNA in VOA supernatants, expressed as replication-competent RNA units per million (RUPM) (24), and by (ii) the detection of HIV P24 in VOA supernatants, expressed as infectious units per million (IUPM) (15) (Fig. 1A).

**FIG 2:**
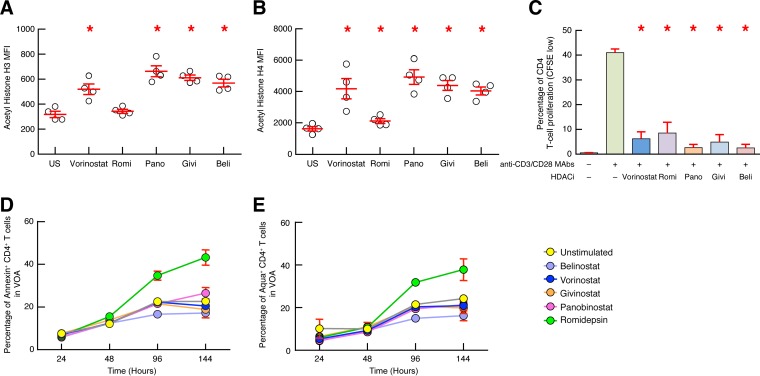
Assessment of functional activity and toxicity of the HDACis tested. (A and B) Mean fluorescence intensity (MFI) of acetyl histone H3 (A) or histone H4 (B). Blood mononuclear cells isolated from 4 HIV-seronegative individuals were exposed or not (unexposed control [US]) to vorinostat (400 nM), romidepsin (Romi [5 nM]), panobinostat (Pano [15 nM]), givinostat (Givi [400 nM]), or belinostat (Beli [400 nM]) for 24 h, and acetyl histone H3 or H4 MFI was analyzed on CD4 T cells by flow cytometry. (C) Percentage of CD4 T-cell proliferation upon HDACi treatment. CFSE-labeled blood mononuclear cells isolated from 3 HIV-seronegative individuals were exposed or not (US) to vorinostat (400 nM), romidepsin (5 nM), panobinostat (15 nM), givinostat (400 nM), or belinostat (400 nM) for 24 h and stimulated for 6 days with anti-CD3/CD28 MAbs, and the percentage of proliferating CD4 T-cell proliferation (CFSE low) was assessed by flow cytometry. The percentage of annexin V-positive (D) or Aqua-positive (E) CD4 T cells was assessed at 24, 48, 96, and 144 h.CD4 T cells isolated from 3 HIV-uninfected individuals were exposed or not to vorinostat (400 nM), romidepsin (5 nM), panobinostat (15 nM), givinostat (400 nM), and belinostat (400 nM) in the presence of allogeneic CD8-depleted PBMCs isolated from HIV-uninfected subjects. Red horizontal lines or histograms correspond to the mean, and red error bars correspond to the SEM. Red asterisks indicate statistical significance (*P* < 0.05) compared to unexposed conditions. Statistical significance (*P* values) was obtained using a one-way ANOVA (Kruskal-Wallis test) followed by a paired *t* test (A to C).

In preliminary experiments, it was determined whether all the HDACis tested, including vorinostat, romidepsin, belinostat, panobinostat, and givinostat, were functionally active. The results obtained showed that all HDACis tested significantly increased acetyl histone H3 and/or H4 mean fluorescent intensity (MFI) (*P* < 0.05) (Fig. 2A and B) and significantly inhibited T-cell receptor (TCR)-induced CD4 T-cell proliferation (*P* < 0.05) (Fig. 2C), thus indicating that all HDACis tested were functionally active. In addition, the viability of CD4 T cells exposed to clinically relevant concentrations of HDACis was also assessed by a flow cytometry-based assay. The results obtained showed that using the aforementioned VOA experimental strategy, all HDACis tested did not significantly influence CD4 T-cell viability compared to that of the unstimulated cells (Fig. 2D and E).

We then evaluated RUPM and IUPM frequencies in 10 aviremic long-term-treated patients (duration of treatment, 2 to 8 years; average, 4.2 years) using the VOA after stimulation with anti-CD3/CD28 MAbs. The cumulative data shown in Fig. 1B through I and Table 1 showed that anti-CD3/CD28 MAb treatment consistently induced HIV-1 replication, as assessed by HIV-1 RNA and P24 production in the culture supernatants, while all replicates (except one at the limit of detection) of the unstimulated condition remained negative for both HIV-1 RNA and P24, demonstrating that the use of allogeneic CD8-depleted blood mononuclear cells from HIV-uninfected subjects in the presence of IL-2 and IL-7 was not sufficient to induce HIV-1 replication and confirmed the results obtained by Bosque et al. (27). In addition, the inducible RUPM and IUPM frequencies measured in the 10 aviremic long-term-treated HIV-1-infected subjects studied were consistent with the frequencies reported in previous studies (15, 24). Of note, the use of anti-CD3/CD28 MAbs to stimulate HIV-1 replication did not underestimate the frequencies of inducible replication-competent virus compared to phytohemagglutin (PHA) stimulation (data not shown).

### HDACis efficiently reactivate HIV-1 replication from latently infected resting memory CD4 T cells isolated from long-term-treated HIV-1-infected subjects.

**FIG 3:**
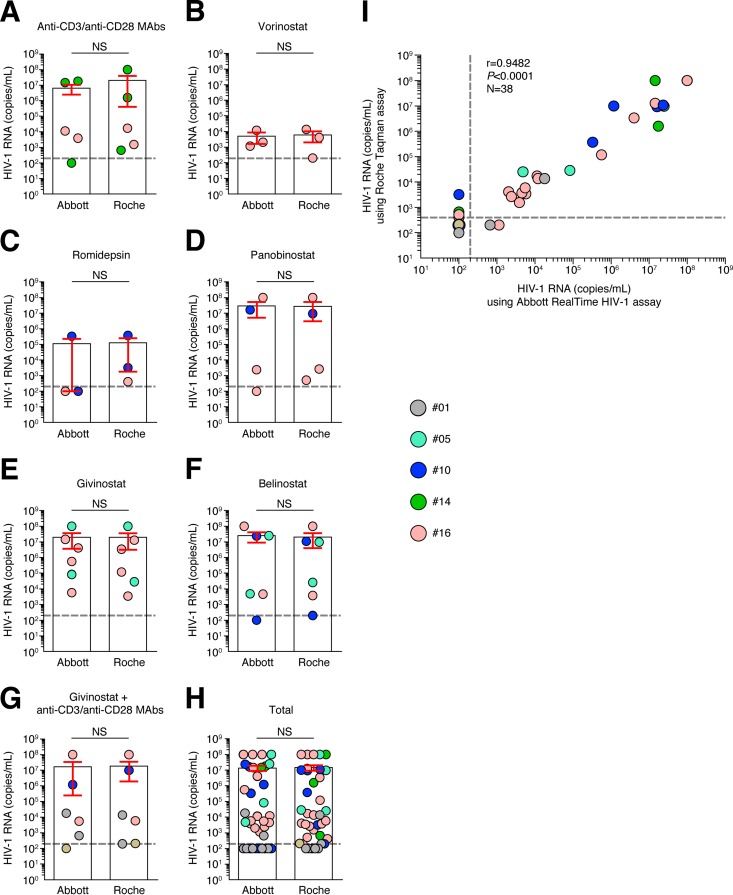
HIV-1 RNA quantification in culture supernatants using Roche TaqMan assay versus the Abbott RealTime HIV-1 assay. Culture supernatants containing a range of HIV-1 RNAs, induced following HDACi treatments, anti-CD3/CD28 MAb treatment, givinostat plus anti-CD3/CD28 MAb treatment, or unexposed cells (*n* = 38) were assessed for HIV-1 RNA using the Roche TaqMan assay (Roche) and Abbott RealTime HIV-1 assay (Abbott). HIV-1 RNA quantification was performed using Roche TaqMan assay versus the Abbott RealTime HIV-1 assay in VOA culture supernatants obtained following anti-CD3/CD28 MAb treatment (A) or treatment with vorinostat (B), romidepsin (C), panobinostat (D), givinostat (E), belinostat (F), or givinostat plus anti-CD3/CD28 MAbs (G). Panel H shows the total results. (I) Correlation between HIV-1 RNA quantification using the Roche TaqMan assay and Abbott RealTime HIV-1. Histograms correspond to the mean, and red error bars correspond to the SEM. NS, not significant. Statistical significance (*P* values) was obtained using Wilcoxon's matched-pair two-tailed signed rank test (A to H) or Spearman's rank correlations (I).

We then evaluated the ability of vorinostat, romidepsin, belinostat, panobinostat, and givinostat to reverse HIV-1 latency using the modified VOA in the 10 aviremic long-term-treated subjects (Table 1). The cumulative data indicated that (i) all of the HDACis tested significantly induced the production of HIV-1 RNA in the culture supernatants compared to untreated cultures, and (ii) the proportions of responder subjects (i.e., subjects positive for HIV-1 RNA in the culture supernatants) were 90% (9 out of 10 subjects) in the cell cultures treated with vorinostat and romidepsin and 100% in cell cultures treated with panobinostat, givinostat, belinostat, and anti-CD3/CD28 (the latter used as the positive control) (*P* < 0.05) (Fig. 1B and D). Of note, no significant differences were observed between the different HDACis tested either in the proportion of subjects with detectable HIV-1 RNA or in the proportion of wells positive for HIV-1 RNA (*P* > 0.05) (Fig. 1B and D). Notably, the use of the Roche TaqMan assay did not overestimate the quantity of HIV-1 RNA present in the culture supernatants, as indicated by the cross-validation test using the Abbott RealTime HIV-1 assay, which avoids the potential detection of HIV-1 DNA (28) (Fig. 3). Indeed, no significant differences (*P* > 0.05) were observed under any condition tested (HDACi treatment and anti-CD3/CD28 MAbs) between the quantification of HIV-1 RNA using the Roche TaqMan assay or the Abbott RealTime HIV-1 assay, demonstrating that the Roche TaqMan assay used in the present study did not overestimate the quantity of HIV-1 RNA present in the culture supernatants under the experimental conditions used.

P24 production was also measured in the cell cultures treated with HDACis compared to HDACi-untreated cell cultures (Fig. 1C and E). Interestingly, anti-CD3/CD28 and givinostat were able to induce P24 in a higher proportion of subjects (i.e., 60 and 50%, respectively) compared to the other HDACis, which induced P24 production in 10 to 30% of subjects (Fig. 1C). Compared to the untreated cultures from the 10 subjects, the proportion of subjects with positive P24 was significantly different (*P* < 0.05) only for anti-CD3/CD28- and givinostat-treated cultures (Fig. 1C). Along the same line, the proportion of wells positive for P24 was significantly higher in anti-CD3/CD28 MAb- and givinostat-treated cultures compared to the other HDACi-treated and -untreated cultures (*P* < 0.05) (Fig. 1E).

With regard to the levels of HIV-1 RNA and P24 measured in the presence of the different HDACis, vorinostat and romidepsin were less efficient at inducing HIV-1 RNA and P24 production than panobinostat, givinostat, and belinostat, which induced levels comparable to anti-CD3/CD28 MAb stimulation (Fig. 1F and G). However, the proportions of P24 responders and P24-positive wells were lower in panobinostat- versus givinostat-treated cultures (Fig. 1C and E). All of the HDACis induced significantly higher levels of HIV-1 RNA and P24 than untreated cell cultures (*P* < 0.05) (Fig. 1F and G).

Finally, the RUPM and IUPM frequencies were then calculated for all conditions. The results indicate that RUPM frequencies induced by all HDACis tested (i.e., vorinostat, romidepsin, panobinostat, givinostat, and belinostat) were significantly increased compared to the frequencies under the unexposed conditions (*P* < 0.05) (Fig. 1H). However, the IUPM frequencies induced by all HDACis tested (i.e., vorinostat, romidepsin, panobinostat, givinostat, and belinostat) were not significantly increased (*P* > 0.05) (Fig. 1I).

**FIG 4:**
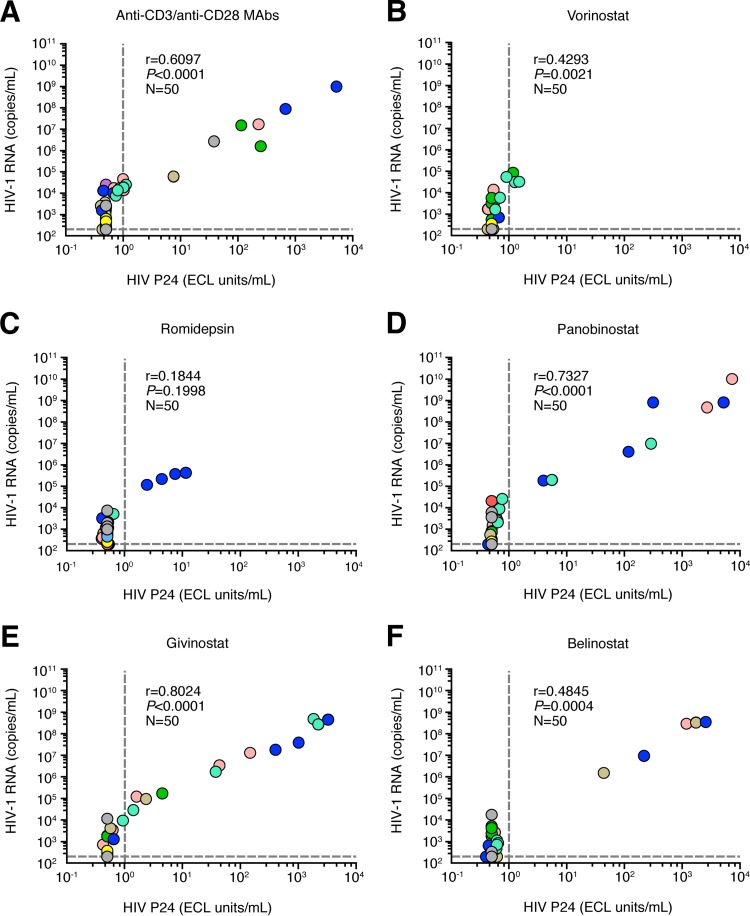
Correlation between P24 and HIV-1 RNA levels detected in culture supernatants following the viral outgrowth assay. Correlation between P24 and HIV-1 RNA levels (*n* = 50; 10 subjects, 5 replicates per condition) was obtained following anti-CD3/CD28 MAb treatment (A) or treatment with vorinostat (B), romidepsin (C), panobinostat (D), givinostat (E), or belinostat (F). Each circle corresponds to one replicate, and each color corresponds to one HIV-1-infected subject. Dotted lines correspond to the limit of detections. Statistical significance (*P* values) was obtained using Spearman's rank correlations.

We then determined the relationship between HIV-1 RNA and P24. These analyses indicated that the levels of P24 production correlated with HIV-1 RNA levels (*r* = 0.59 and *P* < 0.0001) (Fig. 1J). To determine whether HDACis could have differential effect on HIV-1 RNA and P24 production, the levels of HIV-1 RNA and P24 were plotted per treatment condition (Fig. 4A to F). The results obtained showed a direct correlation between P24 and HIV-1 RNA levels in all HDACis tested except following romidepsin exposure (vorinostat, *r* = 0.4293, *P* = 0.0021; romidepsin, *r* = 0.1844, *P* = 0.1998; panobinostat, *r* = 0.7327, *P* < 0.0001; givinostat, *r* = 0.8024, *P* < 0.0001; belinostat, *r* = 0.4845, *P* = 0.0004), suggesting that P24 production induced following HDACi treatments was strongly associated with HIV-1 RNA levels.

### Estimation of the proportion of provirus induced following anti-CD3/CD28 MAb and HDACi treatments.

**FIG 5:**
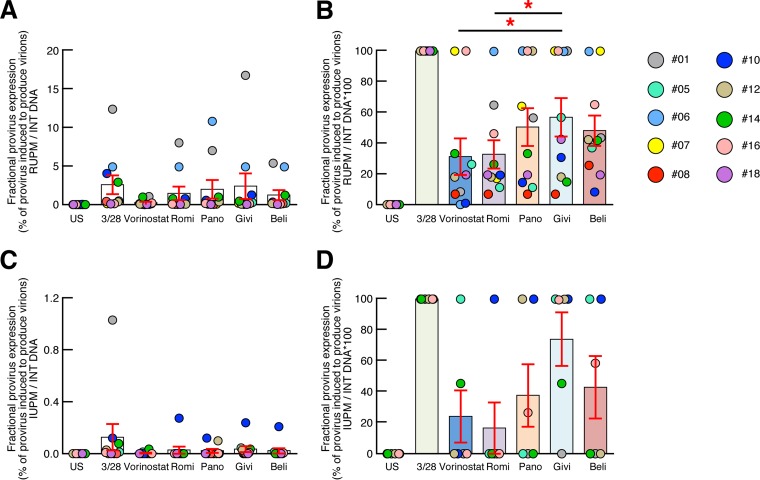
Estimation of the proportion of provirus induced following treatment with anti-CD3/CD28 MAbs and HDACis. (A) Proportion of provirus induced following treatments with anti-CD3/CD28 MAbs and HDACis as assessed by the ratio of RUPM and integrated (INT) DNA (*n* = 10). (B) Proportion of provirus induced following treatment with anti-CD3/CD28 MAbs and HDACis as assessed by the ratio of IUPM and integrated DNA (*n* = 10). (C) Proportion of provirus induced following HDACi treatment compared to treatment with anti-CD3/CD28 MAbs as assessed by RUPM. (D) Proportion of provirus induced following treatment with HDACis compared to anti-CD3/CD28 MAbs as assessed by IUPM (*n* = 6). Red asterisks indicate statistical significance (*P* < 0.05). Statistical significance (*P* values) was obtained using one-way ANOVA (Kruskal-Wallis test) followed by Wilcoxon's matched-pair two-tailed signed rank test.

Characterization of full-length proviruses has illustrated that only 10 to 12% of provirus was inducible, while only a fraction of the provirus was induced following the VOA (29). To estimate the efficiency of HDACis to reverse HIV-1 latency, the proportion of provirus induced by HDACis among total provivus (Fig. 5A and B) or among the proportion of provirus induced by anti-CD3/CD28 MAbs was then estimated (Fig. 5C and D). The results indicate that only a small fraction (≈2.6%) of HIV-1 proviruses were reactivated to produce virions, as assessed by HIV-1 RNA detection following anti-CD3/CD28 MAbs and HDACis (Fig. 5A), supporting the recent results of Cillo et al. (16). However, the fraction of HIV-1 proviruses induced by givinostat represented about 57% of that induced by anti-CD3/CD28 MAb treatment and was significantly higher than the fraction of HIV-1 proviruses induced by vorinostat or romidepsin (Fig. 5B). The fraction of HIV-1 proviruses reactivated to produce virions as assessed by HIV-1 P24 detection following treatment with anti-CD3/CD28 MAbs and HDACis was even lower and represented about 0.13% (Fig. 5C). However, the fraction of HIV-1 proviruses induced by givinostat as assessed by HIV-1 P24 detection represented about 74% of that induced by anti-CD3/CD28 MAb treatment and was higher than the fraction of HIV-1 proviruses induced by all of the other HDACis tested (Fig. 5D), suggesting that givinostat is the most efficient HDACi tested for reversing HIV-1 latency *in vitro*.

### Infectivity of HIV-1 reactivated by HDACi treatment.

**FIG 6:**
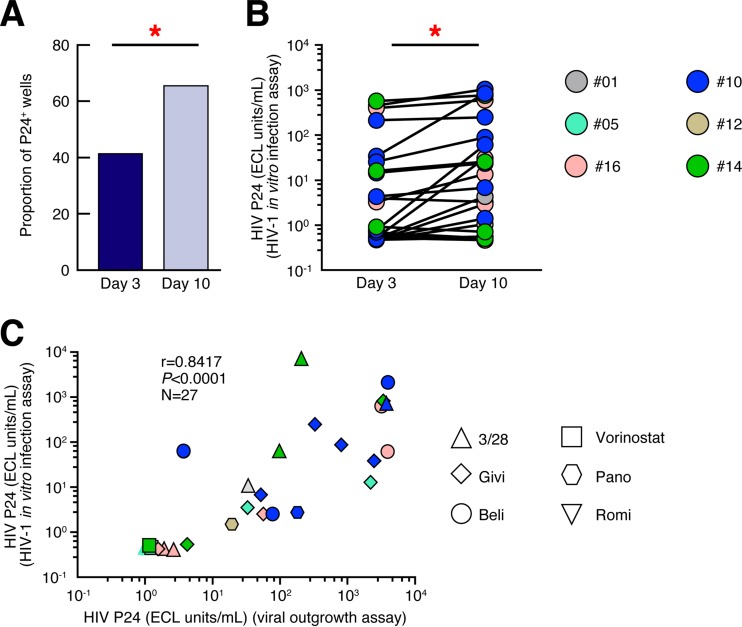
Infectivity of HIV-1 reactivated by HDACi treatment. (A) Proportion of P24-positive wells at days 3 and 10 after *in vitro* HIV-1 inoculation of activated allogeneic CD8-depleted mononuclear cells from HIV-1-negative subjects in the modified VOA culture supernatants (obtained from P24-positive supernatants at day 14) (*n* = 27). (B) P24 values at days 3 and 10 following *in vitro* HIV-1 infection (*n* = 27). (C) Correlation between P24 levels detected in the cell culture supernatants from the *in vitro* infection assay and the P24 levels of the modified VOA supernatants (*n* = 27). Subjects were color coded, and each color corresponds to a subject (B and C). Each condition is depicted with a unique symbol. Red asterisks indicate statistical significance (*P* < 0.05). Statistical significance (*P* values) was obtained using two-tailed chi-square analysis for comparison of positive proportions (A), Wilcoxon's matched-pair two-tailed signed rank test (B), or Spearman's rank correlations (C).

The presence of infectious virus in the culture supernatants of P24-positive cell cultures was then assessed by analyzing the ability of culture supernatants to transmit HIV-1 infection *in vitro*. Activated allogeneic CD8-depleted blood mononuclear cells from HIV-1-uninfected donors were inoculated with supernatants collected from P24-positive cell cultures (termed modified VOA supernatants) of HIV-1-infected subjects for 6 h, and P24 production was determined in the culture supernatants at days 3 and 10 postinoculation by ECL. Of note, P24 was never detected in all culture supernatants at day 0. The cumulative data showed that both the proportion of P24-positive wells and the levels of P24 significantly increased between days 3 and 10 (*P* < 0.05) postinoculation, thus demonstrating that HIV isolated from the modified VOA cell cultures was infectious (Fig. 6A and B). Interestingly, the P24 levels detected in the cell culture supernatants from the new *in vitro* infection correlated with the P24 levels (*r* = 0.8417, *P* < 0.0001) measured in the modified VOA supernatants (Fig. 6C).

### Assessment of potential synergistic effect between givinostat treatment and TCR stimulation or PKC agonist on the reactivation of HIV-1 replication.

**FIG 7:**
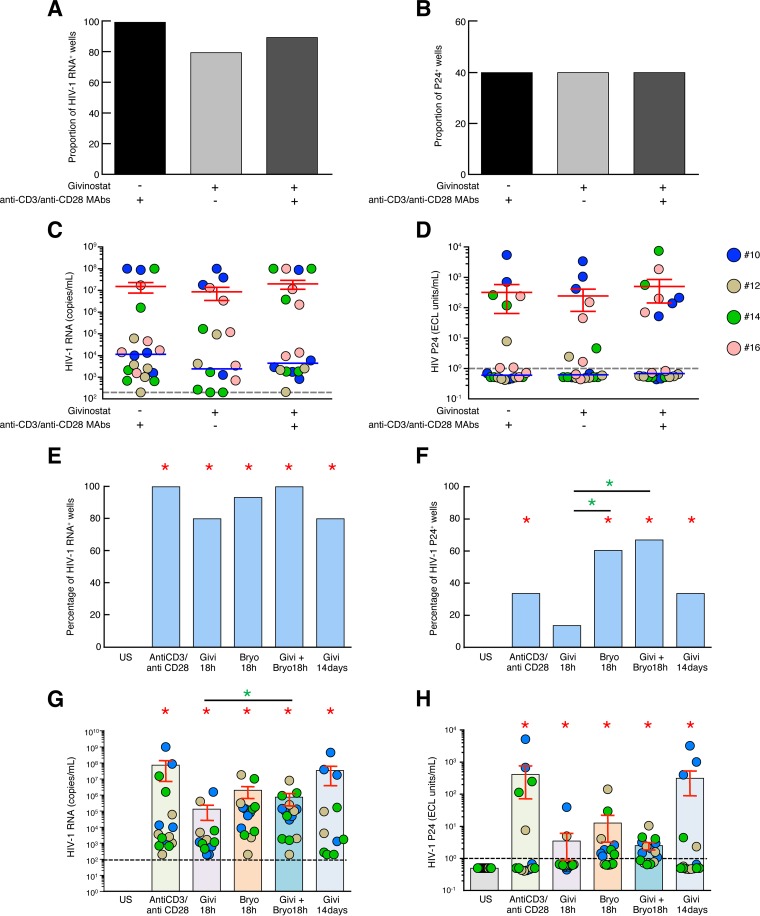
Assessment of potential synergistic effect between givinostat treatment and TCR stimulation or PKC agonist on the reactivation of HIV-1 replication. (A) Proportion of HIV-1 RNA-positive wells following treatment with givinostat and/or anti-CD3/CD28 MAbs (*n* = 4). Wells with detectable HIV-1 RNA (≥200 HIV-1 RNA copies/ml) are indicated as HIV-1 RNA-positive wells for the condition tested. (B) Proportion of P24-positive wells following treatment with givinostat and/or anti-CD3/CD28 MAbs (*n* = 4). Wells with detectable P24 (≥1 ECL unit/ml) are indicated as P24 positive for the condition tested. (C) HIV-1 RNA (copies per milliliter) induced following treatment with givinostat and/or anti-CD3/CD28 MAbs (*n* = 4). (D) Levels of P24 (ECL units per milliliter) induced following treatment with givinostat and/or anti-CD3/CD28 MAbs (*n* = 4). (E) Proportion of HIV-1 RNA-positive wells following treatment with anti-CD3/CD28 MAbs or givinostat (Givi) and/or bryostatin (Bryo) (*n* = 3). Wells with detectable HIV-1 RNA (≥200 HIV-1 RNA copies/ml) are indicated as HIV-1 RNA-positive wells for the condition tested. US, unstimulated. (F) Proportion of P24-positive wells following treatment with anti-CD3/CD28 MAbs or givinostat and/or bryostatin (*n* = 3). Wells with detectable P24 (≥1 ECL unit/ml) are indicated as P24-positive wells for the condition tested. (G) Levels of HIV-1 RNA copies per milliliter induced following treatment with anti-CD3/CD28 MAbs or givinostat and/or bryostatin (*n* = 3). (H) Levels of HIV-1 P24 (ECL units per milliliter) induced following treatment with anti-CD3/CD28 MAbs or givinostat and/or bryostatin (*n* = 3). Subjects were color coded, and each color corresponds to a subject (C and D and G and H). Red error bars correspond to means ± SEM. Red asterisks indicate statistical significance (*P* < 0.05). Green asterisks indicate statistical significance compared to the givinostat (18-h) condition (*P* < 0.05). Statistical significance (*P* values) was obtained using two-tailed chi-square analysis for comparison of positive proportions (A, B, E, and F) or by one-way ANOVA (Kruskal-Wallis test) followed by Wilcoxon matched-pair two-tailed signed rank test (C, D, G, and H).

To evaluate whether the HDACis and TCR stimulation may synergize in the reactivation of HIV-1 replication, resting memory CD4 T cells isolated from aviremic long-term-treated HIV-1-infected subjects known to be P24 positive following givinostat treatment were exposed to givinostat or anti-CD3/CD28 MAbs alone or in combination. The cumulative data showed that neither the proportion of HIV-1 RNA/P24-positive wells (Fig. 7A and B) nor the levels of HIV-1 RNA/P24 (Fig. 7C and D) significantly increased upon the combined anti-CD3/CD28 MAb–givinostat treatment (*P* > 0.05). These results suggested that both HDACis and TCR signals may target similar populations of latently HIV-1-infected resting memory CD4 T cells.

The study by Laird et al. has recently demonstrated that combination of mechanistically distinct LRAs may synergize to induce HIV-1 transcription (18). To address whether givinostat may synergize with bryostatin (a protein kinase C [PKC] agonist), resting memory CD4 T cells were isolated from 3 long-term-treated aviremic HIV-infected subjects and cultured in the presence of allogeneic CD8-depleted PBMCs. Cells were then exposed to givinostat or bryostatin alone or in combination for 18 h. As an internal control, cells were also exposed to givinostat for 14 days. HIV replication was assessed at day 14 of the VOA by the detection of HIV-1 RNA and P24 in the culture supernatants. The cumulative data showed that under all conditions tested viral replication was significantly reactivated compared to under unstimulated culture conditions, as assessed by the proportion of HIV-1 RNA/P24-positive wells or by the levels of HIV-1 RNA/P24 (*P* < 0.05) (Fig. 7E to H). Interestingly, the combined givinostat-bryostatin treatment significantly increased the levels of HIV-1 RNA/P24 compared to cells exposed to givinostat alone for 18 h. However, the levels of HIV RNA/P24 produced in the combined givinostat-bryostatin treatment were not significantly higher than the levels of HIV RNA/P24 produced in the culture supernatants of cells exposed to bryostatin alone or those with prolonged/repeated exposure to givinostat (*P* > 0.05) (Fig. 7E to H). Taken together, these results suggest that givinostat may synergize with mechanistically different LRAs such as bryostatin. However, further investigations may be required.

### Prolonged/repeated treatment of resting memory CD4 T cells to HDACis is the primary mechanism responsible for efficient induction of HIV-1 replication by HDACi.

Finally, we evaluated (i) the contribution of prolonged/repeated treatment (i.e., 14 days) of resting memory CD4 T cells to HDACis and (ii) the role of allogeneic CD8-depleted blood mononuclear cells in the modified VOA as a source of potential target cells for HIV-1 and/or as the provider of an activation signal.

**FIG 8:**
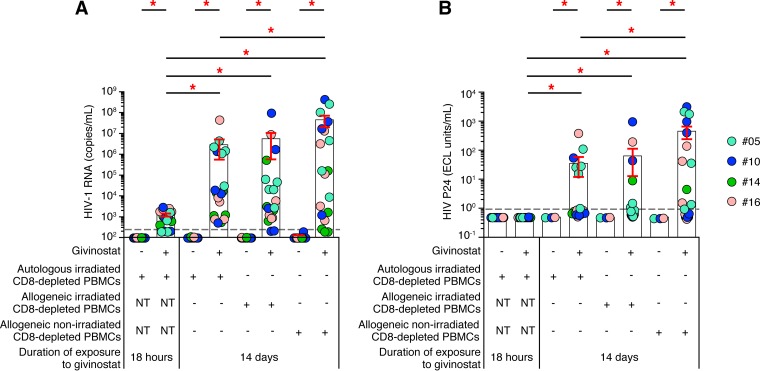
Prolonged/repeated exposure of resting memory CD4 T cells to HDACis is the primary mechanism responsible for efficient induction of HIV-1 replication by HDACi. (A) Levels of HIV-1 RNA (copies per milliliter) induced following givinostat treatment (*n* = 4; 5 replicates). (B) Levels of P24 (ECL units per milliliter) induced following givinostat treatment (*n* = 4; 5 replicates). Subjects were color coded, and each color corresponds to a subject. US, unstimulated or unexposed. Histograms correspond to the mean, and red error bars correspond to the SEM. Red asterisks indicate statistical significance (*P* < 0.05). Statistical significance (*P* values) was obtained using one-way ANOVA (Kruskal-Wallis test) followed by Wilcoxon's matched-pair two-tailed signed rank test.

To address the first issue, resting memory CD4 T cells isolated from aviremic long-term-treated HIV-1-infected subjects were treated with givinostat for 18 h or 14 days in the presence of autologous irradiated PBMCs. The use of autologous irradiated CD8-depleted PBMCs prevented the increase of transcriptional noise induced by mixed leukocyte reaction (MLR), and the fact that the cells were irradiated prevented the amplification of the virus through viral spreading. The results clearly indicate that the treatment with givinostat for 14 days induced significantly higher levels of HIV-1 RNA and P24 in the culture supernatants than in the culture treated for 18 h (*P* < 0.05) (Fig. 8A). Therefore, prolonged treatment with HDACis is a powerful strategy to break HIV-1 latency in resting memory CD4 T cells from long-term aviremic ART-treated patients *in vitro*.

**FIG 9:**
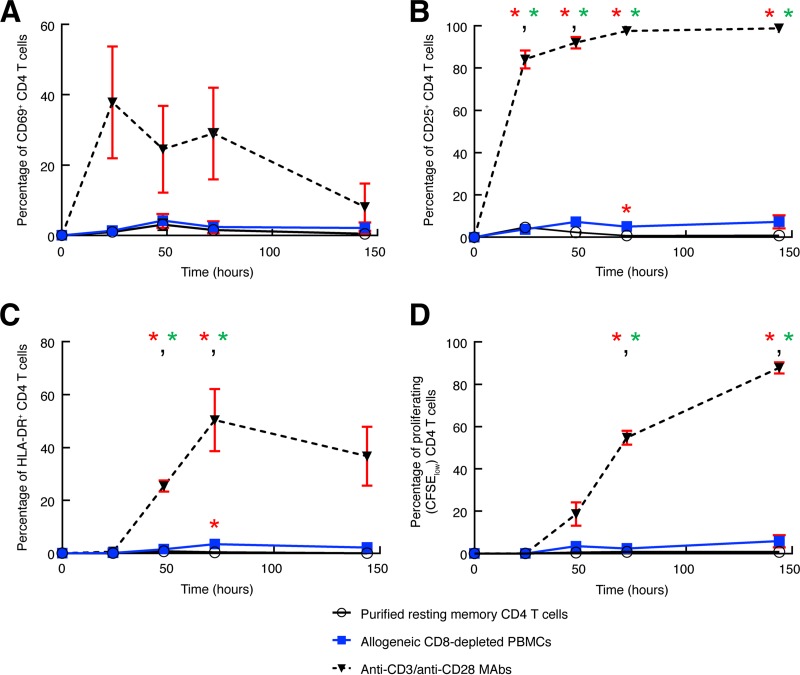
Level of expression of activation markers and proliferation capacity of purified resting memory CD4 T cells exposed to allogeneic CD8-depleted mononuclear cells. (A to C) Percentages of CD69 (early) (A), CD25 (intermediate) (B), and HLA-DR (late) (C) activation marker expression on purified resting memory CD4 T cells (*n* = 3) following 0, 1, 2, 3, and 6 days of exposure to allogeneic CD8-depleted blood mononuclear cells or to anti-CD3/CD28 MAbs (positive control). Unexposed resting memory CD4 T cells were used as a negative control. (D) Percentage of proliferating resting memory CD4 T cells (CFSE_low_ [*n* = 3]) following 0, 1, 2, 3, and 6 days of exposure to allogeneic CD8-depleted mononuclear cells or to anti-CD3/CD28 MAbs (positive control). Unexposed resting memory CD4 T cells were used as a negative control. Red error bars correspond to the mean ± SEM. Red asterisks indicate statistical significance compared to unstimulated (US) (*P* < 0.05). Green asterisks indicate statistical significance compared to allogeneic CD8-depleted PBMCs (MLR) condition (*P* < 0.05). Statistical significance (*P* values) was obtained using paired Student's *t* tests.

With regard to the second issue, resting memory CD4 T cells isolated from aviremic long-term-treated HIV-1-infected subjects were treated with givinostat for 14 days in the presence of allogeneic irradiated or allogeneic nonirradiated CD8-depleted PBMCs, and levels of HIV-1 RNA and P24 were compared to those found in the cultures containing autologous irradiated CD8-depleted PBMCs. In this context, the use of allogeneic irradiated CD8-depleted PBMCs allowed the evaluation of the impact of the increase of transcriptional noise induced by MLR and prevented the amplification of the virus through viral spreading. Finally, the use of allogeneic nonirradiated CD8-depleted PBMCs allowed the evaluation of both the increase of transcriptional noise induced by MLR and the signal amplification through viral spreading. No significant differences (*P* > 0.05) were observed in the levels of HIV-1 RNA and P24 in the culture supernatants of cultures treated with givinostat for 14 days in the presence of autologous or allogeneic irradiated CD8-depleted PBMCs (Fig. 8A and B). These results further confirm the importance of the prolonged treatment of HDACis as the primary mechanism responsible for the induction of HIV-1 replication in resting memory CD4 T cells. However, a slight significant increase (*P* < 0.05) in the levels of HIV-1 RNA and P24 was found in the culture supernatants of the allogeneic CD8-depleted nonirradiated PBMCs compared to autologous irradiated CD8-depleted PBMC cultures (Fig. 8A and B). The results suggested that using allogeneic CD8-depleted nonirradiated PBMCs may be associated with some degree of cell activation that can influence the amplification of HIV in the cell cultures. In this regard, it was observed that the use of allogeneic nonirradiated CD8-depleted PBMCs was associated with a moderate increase in the percentage of activated and proliferating CD4 T cells, as assessed by CD25 and HLA-DR expression (5.1% versus 0.8% and 3.5% versus 0.4% at day 3 in the allogeneic nonirradiated and allogeneic irradiated cell cultures, respectively) (Fig. 9) and by CFSE-based assay (5.1% versus 0.8% at day 6; *P* > 0.05) (Fig. 9).

## DISCUSSION

Systematic preclinical evaluation of potential LRAs in primary CD4 T cells isolated from HIV-infected individuals on ART represents a fundamental step to advance toward clinical trials. Evidence that LRAs reactivate HIV-1 replication from primary resting memory CD4 T cells isolated from aviremic long-term-treated HIV-1-infected subjects is limited and remains challenging, while recent clinical trials based on vorinostat, romidepsin, and panobinostat performed in HIV-1 patients under ART showed modest but encouraging effects (20, 26; O. S. Sogaard, presented at AIDS 2014: 20th International AIDS Conference, Melbourne, Australia [www.aids2014.org]).

In the present study, we hypothesized that increasing the duration of exposure of resting memory CD4 T cells to HDACis may result in efficient induction and amplification of HIV-1 replication in resting memory CD4 T cells of aviremic long-term-treated HIV-1-infected subjects *in vitro*. The bulk of the data presented in Fig. 1, 3, 4, and 5 were generated using a 14-day treatment of cultures with the different HDACis in combination with allogeneic nonirradiated CD8-depleted PBMCs. The individual contributions of the modifications implemented in the newly developed VOA were also assessed.

The presence of HIV-1 RNA and P24 was measured in VOA culture supernatants using validated diagnostic assays and allowed the assessment of (i) the proportion of responders, (ii) the proportion of positive wells, (iii) the levels of HIV-1 RNA and P24, and (iv) the RUPM and IUPM frequencies, as well as (v) determination of the proportion of provirus induced following anti-CD3/CD28 MAb and HDACi treatments. The results first indicated that the signal induced by allogeneic stimulation was not sufficient to induce HIV-1 replication, as assessed by HIV-1 RNA and P24 detection in the culture supernatants, since all replicates (except one at the limit of detection) of the unstimulated condition remained negative for both HIV-1 RNA and P24. Interestingly, the results indicated that vorinostat and romidepsin were less efficient at inducing HIV-1 RNA and P24 production than panobinostat, givinostat, and belinostat. Of note, the results obtained with panobinostat mirror the results obtained *in vivo* by Rasmussen et al., which showed that repeated exposure to panobinostat effectively disrupts HIV-1 latency *in vivo* (20). Interestingly, givinostat was more efficient at inducing P24 production than vorinostat, panobinostat, and romidepsin at reversing HIV-1 latency *in vitro*.

Characterization of full-length proviruses has illustrated that only 10 to 12% of proviruses were inducible, while only a fraction of them were induced following VOA (29). Interestingly, the reactivated provirus induced by givinostat represented 57 to 74% of that induced by anti-CD3/CD28 MAb treatment and was significantly higher than the fraction of HIV-1 proviruses induced by vorinostat or romidepsin. In addition, the combined treatment of resting CD4 T cells with givinostat and anti-CD3/CD28 MAbs did not significantly increase HIV-1 replication compared to givinostat treatment alone, suggesting that givinostat and TCR signals may target similar populations of latently HIV-1-infected resting memory CD4 T cells and confirmed the proportion of reactivated provirus induced by givinostat. Similarly, the combined treatment of resting CD4 T cells with givinostat and bryostatin (a PKC agonist) did not significantly increase HIV-1 replication compared to bryostatin treatment alone, suggesting that givinostat may synergize with mechanistically different LRAs such as bryostatin. However, further investigations may be required.

Of note, givinostat has not been evaluated in an HIV clinical trial but is currently being evaluated in a phase II clinical trial for leukemia and has been approved by the European Union for treatment of juvenile arthritis since 2010 (30). Givinostat has been shown to be safe upon single or repeated oral dose administration, is eliminated slowly (half-life of 6 to 7 h), and shows robust dose-dependent pharmacokinetics up to a dose of 600 mg daily, achieving a maximum concentration of 542 ± 93 ng/ml in the blood of healthy individuals. Similar to its short-term use, there have been no adverse effects associated with long-term use of givinostat (30). Its administration has been associated with mild but reversible effects on platelet and neutrophil levels in blood and with a transient decrease in the levels of proinflammatory cytokine production (30).

We have also demonstrated that HIV-1 reactivated in the VOA cell culture supernatants was not only replication competent but also infectious using an *in vitro* HIV-1 infection assay. In addition, the P24 levels detected in the cell culture supernatants from the new *in vitro* infection correlated with the P24 levels measured in the modified VOA supernatants regardless of the original VOA treatment, thus suggesting that the presence of infectious particles was directly associated with the efficiency of the reactivation.

Finally, we have evaluated the contribution of the parameters modified in the present VOA: i.e., (i) prolonged/repeated HDACi treatment and (ii) use of allogeneic CD8-depleted blood mononuclear cells as a provider of an activation signal and/or as a source of *de novo* CD4 T-cell targets for HIV-1. We provide evidence that the prolonged treatment with HDACis is the primary mechanism responsible for the reactivation of HIV-1 replication in resting memory CD4 T cells of aviremic long-term-ART-treated patients. The use of allogeneic nonirradiated blood mononuclear cells appears to have a secondary effect since it was associated with only a moderate increase in the percentage of activated CD4 T cells and a minor effect on HIV-1 replication.

In summary, the present modified VOA represents a powerful tool in order to evaluate consistently the potency of novel LRAs and/or combination therapies to reactivate HIV-1 replication in resting memory CD4 T cells of long-term-treated aviremic HIV-1 infected subjects. Using this assay, we demonstrated that prolonged/repeated treatment of resting memory CD4 T cells with HDACis induced HIV-1 replication and production from primary resting memory CD4 T cells isolated from aviremic long-term-treated HIV-1-infected subjects. Interestingly, the viral particles produced were not only replication competent but also infectious. Notably, givinostat, an HDACi that has not been investigated in clinical trials, was more efficient than vorinostat, panobinostat, and romidepsin in reversing HIV-1 latency *in vitro*. The reason why givinostat harbors enhanced efficacy of latency reversal *in vitro* remains to be established. However, the fact that givinostat was used at a higher concentration (i.e., 400 nM) than panobinostat and romidepsin might have contributed to its enhanced *in vitro* efficacy of latency reversal. Indeed, the concentrations of panobinostat and romidepsin used in our experimental settings were about 26- and 80-fold lower than that of givinostat. Of note, all HDACis were used at clinically relevant concentrations.

Taken together, these results support further evaluation of givinostat as a latency-reversing agent in aviremic long-term-treated HIV-1-infected subjects.

## References

[1] Chun TW, Stuyver L, Mizell SB, Ehler LA, Mican JA, Baseler M, Lloyd AL, Nowak MA, Fauci AS 1997 Presence of an inducible HIV-1 latent reservoir during highly active antiretroviral therapy. Proc Natl Acad Sci U S A 94:13193–13197. doi:10.1073/pnas.94.24.13193. 9371822 PMC24285

[2] Chun TW, Carruth L, Finzi D, Shen X, DiGiuseppe JA, Taylor H, Hermankova M, Chadwick K, Margolick J, Quinn TC, Kuo YH, Brookmeyer R, Zeiger MA, Barditch-Crovo P, Siliciano RF 1997 Quantification of latent tissue reservoirs and total body viral load in HIV-1 infection. Nature 387:183–188. doi:10.1038/387183a0. 9144289

[3] Finzi D, Hermankova M, Pierson T, Carruth LM, Buck C, Chaisson RE, Quinn TC, Chadwick K, Margolick J, Brookmeyer R, Gallant J, Markowitz M, Ho DD, Richman DD, Siliciano RF 1997 Identification of a reservoir for HIV-1 in patients on highly active antiretroviral therapy. Science 278:1295–1300. doi:10.1126/science.278.5341.1295. 9360927

[4] Wong JK, Hezareh M, Gunthard HF, Havlir DV, Ignacio CC, Spina CA, Richman DD 1997 Recovery of replication-competent HIV despite prolonged suppression of plasma viremia. Science 278:1291–1295. doi:10.1126/science.278.5341.1291. 9360926

[5] Siliciano JD, Kajdas J, Finzi D, Quinn TC, Chadwick K, Margolick JB, Kovacs C, Gange SJ, Siliciano RF 2003 Long-term follow-up studies confirm the stability of the latent reservoir for HIV-1 in resting CD4^+^ T cells. Nat Med 9:727–728. doi:10.1038/nm880. 12754504

[6] Chomont N, El-Far M, Ancuta P, Trautmann L, Procopio FA, Yassine-Diab B, Boucher G, Boulassel MR, Ghattas G, Brenchley JM, Schacker TW, Hill BJ, Douek DC, Routy JP, Haddad EK, Sekaly RP 2009 HIV reservoir size and persistence are driven by T cell survival and homeostatic proliferation. Nat Med 15:893–900. doi:10.1038/nm.1972. 19543283 PMC2859814

[7] Deeks SG 2012 HIV: shock and kill. Nature 487:439–440. doi:10.1038/487439a. 22836995

[8] Wightman F, Ellenberg P, Churchill M, Lewin SR 2012 HDAC inhibitors in HIV. Immunol Cell Biol 90:47–54. doi:10.1038/icb.2011.95. 22083528

[9] Rasmussen TA, Schmeltz Sogaard O, Brinkmann C, Wightman F, Lewin SR, Melchjorsen J, Dinarello C, Ostergaard L, Tolstrup M 2013 Comparison of HDAC inhibitors in clinical development: effect on HIV production in latently infected cells and T-cell activation. Hum Vaccin Immunother 9:993–1001. doi:10.4161/hv.23800. 23370291 PMC3899169

[10] Shan L, Xing S, Yang HC, Zhang H, Margolick JB, Siliciano RF 2013 Unique characteristics of histone deacetylase inhibitors in reactivation of latent HIV-1 in Bcl-2-transduced primary resting CD4^+^ T cells. J Antimicrob Chemother 69:28–33. doi:10.1093/jac/dkt338. 23999005 PMC3861332

[11] Archin NM, Espeseth A, Parker D, Cheema M, Hazuda D, Margolis DM 2009 Expression of latent HIV induced by the potent HDAC inhibitor suberoylanilide hydroxamic acid. AIDS Res Hum Retroviruses 25:207–212. doi:10.1089/aid.2008.0191. 19239360 PMC2853863

[12] Sahu GK, Cloyd MW 2011 Latent HIV in primary T lymphocytes is unresponsive to histone deacetylase inhibitors. Virol J 8:400. doi:10.1186/1743-422X-8-400. 21838863 PMC3168425

[13] Saleh S, Wightman F, Ramanayake S, Alexander M, Kumar N, Khoury G, Pereira C, Purcell D, Cameron PU, Lewin SR 2011 Expression and reactivation of HIV in a chemokine induced model of HIV latency in primary resting CD4^+^ T cells. Retrovirology 8:80. doi:10.1186/1742-4690-8-80. 21992606 PMC3215964

[14] Blazkova J, Chun TW, Belay BW, Murray D, Justement JS, Funk EK, Nelson A, Hallahan CW, Moir S, Wender PA, Fauci AS 2012 Effect of histone deacetylase inhibitors on HIV production in latently infected, resting CD4(+) T cells from infected individuals receiving effective antiretroviral therapy. J Infect Dis 206:765–769. doi:10.1093/infdis/jis412. 22732922 PMC3491743

[15] Bullen CK, Laird GM, Durand CM, Siliciano JD, Siliciano RF 2014 New ex vivo approaches distinguish effective and ineffective single agents for reversing HIV-1 latency in vivo. Nat Med 20:425–429. doi:10.1038/nm.3489. 24658076 PMC3981911

[16] Cillo AR, Sobolewski MD, Bosch RJ, Fyne E, Piatak M Jr, Coffin JM, Mellors JW 2014 Quantification of HIV-1 latency reversal in resting CD4^+^ T cells from patients on suppressive antiretroviral therapy. Proc Natl Acad Sci U S A 111:7078–7083. doi:10.1073/pnas.1402873111. 24706775 PMC4024870

[17] Ylisastigui L, Archin NM, Lehrman G, Bosch RJ, Margolis DM 2004 Coaxing HIV-1 from resting CD4 T cells: histone deacetylase inhibition allows latent viral expression. AIDS 18:1101–1108. doi:10.1097/00002030-200405210-00003. 15166525

[18] Laird GM, Bullen CK, Rosenbloom DI, Martin AR, Hill AL, Durand CM, Siliciano JD, Siliciano RF 2015 Ex vivo analysis identifies effective HIV-1 latency-reversing drug combinations. J Clin Invest 125:1901–1912. doi:10.1172/JCI80142. 25822022 PMC4463209

[19] Archin NM, Bateson R, Tripathy MK, Crooks AM, Yang KH, Dahl NP, Kearney MF, Anderson EM, Coffin JM, Strain MC, Richman DD, Robertson KR, Kashuba AD, Bosch RJ, Hazuda DJ, Kuruc JD, Eron JJ, Margolis DM 2014 HIV-1 expression within resting CD4^+^ T cells after multiple doses of vorinostat. J Infect Dis 210:728–735. doi:10.1093/infdis/jiu155. 24620025 PMC4148603

[20] Rasmussen TA, Tolstrup M, Brinkmann CR, Olesen R, Erikstrup C, Solomon A, Winckelmann A, Palmer S, Dinarello C, Buzon M 2014 Panobinostat, a histone deacetylase inhibitor, for latent-virus reactivation in HIV-infected patients on suppressive antiretroviral therapy: a phase 1/2, single group, clinical trial. Lancet HIV 1:e13–e21. doi:10.1016/S2352-3018(14)70014-1. 26423811

[21] Dar RD, Hosmane NN, Arkin MR, Siliciano RF, Weinberger LS 2014 Screening for noise in gene expression identifies drug synergies. Science 344:1392–1396. doi:10.1126/science.1250220. 24903562 PMC4122234

[22] Perreau M, Kremer EJ 2005 Frequency, proliferation, and activation of human memory T cells induced by a nonhuman adenovirus. J Virol 79:14595–14605. doi:10.1128/JVI.79.23.14595-14605.2005. 16282459 PMC1287557

[23] Vandergeeten C, Fromentin R, Merlini E, Lawani MB, DaFonseca S, Bakeman W, McNulty A, Ramgopal M, Michael N, Kim JH, Ananworanich J, Chomont N 2014 Cross-clade ultrasensitive PCR-based assays to measure HIV persistence in large-cohort studies. J Virol 88:12385–12396. doi:10.1128/JVI.00609-14. 25122785 PMC4248919

[24] Sobolewski M, Cillo A, Lalama CM, Bosch RJ, Mellors J 2013 A rapid virion recovery assay reveals a larger population of inducible proviruses in resting CD4^+^ T-cells than previously recognized, abstr 174LB Conf Retrovir Opportunistic Infect, 3 to 6 March, Atlanta, GA.

[25] Hu Y, Smyth GK 2009 ELDA: extreme limiting dilution analysis for comparing depleted and enriched populations in stem cell and other assays. J Immunol Methods 347:70–78. doi:10.1016/j.jim.2009.06.008. 19567251

[26] Archin NM, Liberty AL, Kashuba AD, Choudhary SK, Kuruc JD, Crooks AM, Parker DC, Anderson EM, Kearney MF, Strain MC, Richman DD, Hudgens MG, Bosch RJ, Coffin JM, Eron JJ, Hazuda DJ, Margolis DM 2012 Administration of vorinostat disrupts HIV-1 latency in patients on antiretroviral therapy. Nature 487:482–485. doi:10.1038/nature11286. 22837004 PMC3704185

[27] Bosque A, Famiglietti M, Weyrich AS, Goulston C, Planelles V 2011 Homeostatic proliferation fails to efficiently reactivate HIV-1 latently infected central memory CD4^+^ T cells. PLoS Pathog 7:e1002288. doi:10.1371/journal.ppat.1002288. 21998586 PMC3188522

[28] Cloherty G, Swanson P, Lucic D, Dieckhaus K, Anthony P, Cataline P, Herman C, Hackett J Jr, Skolnik PR, Chirch L 2014 Clinical implications of elevated HIV-1 viral load results obtained from samples stored frozen in Vacutainer plasma preparation tubes. J Virol Methods 204:91–92. doi:10.1016/j.jviromet.2014.01.025. 24747107

[29] Ho YC, Shan L, Hosmane NN, Wang J, Laskey SB, Rosenbloom DI, Lai J, Blankson JN, Siliciano JD, Siliciano RF 2013 Replication-competent noninduced proviruses in the latent reservoir increase barrier to HIV-1 cure. Cell 155:540–551. doi:10.1016/j.cell.2013.09.020. 24243014 PMC3896327

[30] Furlan A, Monzani V, Reznikov LL, Leoni F, Fossati G, Modena D, Mascagni P, Dinarello CA 2011 Pharmacokinetics, safety and inducible cytokine responses during a phase 1 trial of the oral histone deacetylase inhibitor ITF2357 (givinostat). Mol Med 17:353–362. doi:10.2119/molmed.2011.00020. 21365126 PMC3105139

[31] Søgaard OS, Graversen ME, Leth S, Olesen R, Brinkmann CR, Nissen SK, Kjaer AS, Schleimann MH, Denton PW, Hey-Cunningham WJ, Koelsch KK, Pantaleo G, Krogsgaard K, Sommerfelt M, Fromentin R, Chomont N, Rasmussen TA, Østergaard L, Tolstrup M 2015 The depsipeptide romidepsin reverses HIV-1 latency *in vivo*. PLoS Pathog 11:e1005142. doi:10.1371/journal.ppat.1005142. PMC4575032 26379282

